# Halbach Array Induced Magnetic Field Alignment in Boron Nitride Nanocomposites

**DOI:** 10.1002/advs.202408532

**Published:** 2024-12-23

**Authors:** Viney Ghai, Ases Akas Mishra, Enling Huang, Roselle Ngaloy, Saroj P. Dash, Aleksandar Matic, Roland Kádár

**Affiliations:** ^1^ Department of Industrial and Materials Science Chalmers University of Technology Göteborg 41296 Sweden; ^2^ Department of Microtechnology and Nanoscience Chalmers University of Technology Göteborg 41296 Sweden; ^3^ Department of Physics Chalmers University of Technology Göteborg 41296 Sweden

**Keywords:** actuator, alignment, boron nitride, Halbach array, thermo‐electric enhancement

## Abstract

Thermal conductivity enhancement in polymers is vital for advanced applications. This study introduces a novel method to align hexagonal boron nitride (hBN) nanosheets within polydimethylsiloxane (PDMS) matrices using a Halbach array to create a highly uniform magnetic field. This technique achieves significant improvements in thermal conductivity by effectively aligning hBN nanosheets. This research shows that hBN nanosheets, when aligned, can drastically enhance thermal conductivity in PDMS composites. Specifically, 10 wt.% vertically aligned hBN nanosheets in a rotating magnetic field achieve a thermal conductivity of 3.58 W mK^−1^, an impressive 1950% increase over pure PDMS. Additionally, the study explores the effects of orientation on dielectric properties, finding that the orientation of hBN nanosheets also improves electrical insulation and increases the dielectric constant while maintaining extremely low dielectric losses. For a vertically oriented sample, the dielectric constant reaches ≈14, and dielectric losses are as low as 0.0049 at 100 Hz, highlighting their potential for energy storage capacitors. This approach not only enhances thermal management but also maintains or improves electrical insulation, offering promising advances for polymer composites in various technological applications.

## Graphical abstract

This study examines the magnetic field alignment of hBN in polymer matrices using a Halbach array. Aligning 10 wt.% hBN within PDMS achieves notable thermal conductivity improvements, reaching up to 3.58 W mK^−1^. The composites also maintain high electrical insulation, enhanced dielectric properties, and potential functionality as magnetic actuators, enabling applications in energy storage, electronics, and heat management.

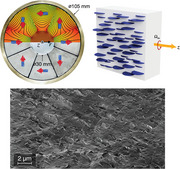

## 1 Introduction

Over the past few decades, researchers have been captivated by the quest to enhance the thermal conductivity of polymers. This pursuit stems from the critical need for materials with improved thermal management properties across various industries, including electronics, aerospace, automotive, and biomedical fields. Despite significant strides, achieving thermal conductivity in polymers comparable to metals remains a formidable challenge, limiting their widespread adoption in high‐performance applications. Efforts to boost thermal conductivity in insulating polymers have predominantly centered around incorporating various conductive fillers.^[^ ^1^, ^2^ ^]^ These fillers, including carbon nanotubes (CNTs), carbon black, graphite, boron nitride, and graphene nanosheets, have been explored extensively.^[^ ^1^, ^3^, ^4^ ^]^ Among these options, boron nitride (BN) has emerged as a notable filler due to its remarkable capacity to enhance thermal conductivity.^[^ ^5^ ^]^ Unlike graphene, higher tendency to agglomerate within polar polymer matrices due to its excellent electrical conductivity and strong π–π stacking interactions between graphene layers. hBN exhibits superior dispersion in polar polymers, thus offering promising results for thermal enhancement applications. Boron nitride (BN) is a versatile material with unique thermal properties that make it highly sought after for various thermal applications. As a compound of boron and nitrogen, hBN exists in several crystalline forms, including hexagonal and cubic structures, each with distinct properties.^[^ ^6^ ^]^ BN's exceptional thermal conductivity, comparable to that of diamond, alongside its excellent thermal stability and resistance to chemical reactions, renders it an ideal candidate for thermal management applications. In particular, BN's ability to efficiently conduct heat, even at high temperatures, makes it invaluable for use in thermal interface materials, where it can effectively dissipate heat away from electronic components to prevent overheating and improve device reliability.^[^ ^7^ ^]^ Among the many ceramics materials, hBN nanosheets have gained significant interest due to their anisotropic thermal conductivity, with values of ≈600 W mK^−1^ (in‐plane) and ≈30 W mK^−1^ (out‐of‐plane).^[^ ^8^ ^]^ Additionally, hBN's thermal insulation properties find applications in industries such as aerospace and automotive, where it is utilized to protect sensitive equipment from extreme temperatures. Overall, hBN versatility and exceptional thermal characteristics position it as a key material for addressing thermal challenges across various fields, from electronics to aerospace engineering.^[^ ^6^ ^]^ The importance of hBN nanofillers' enhancing thermal conductivity in polymers extends beyond mere heat dissipation. It influences critical properties such as mechanical strength, barrier performance, antibacterial properties and flame retardancy, making it a pivotal parameter for a wide range of applications.^[^ ^9^, ^10^, ^11^, ^12^ ^]^ These multifaceted enhancements position boron nitride‐polymer composites as promising candidates for addressing diverse technological challenges. Despite significant advancements, challenges persist in optimizing the dispersion and alignment of hBN within polymer matrices to fully harness its potential for enhancing thermal conductivity. Addressing these challenges necessitates interdisciplinary approaches that integrate materials science, polymer chemistry, and engineering principles. Several methods have emerged as potential solutions for enhancing thermal conductivity by aligning hBN within polymers, including shear flow and magnetic field orientation.^[^ ^5^, ^13^ ^]^

As we have recently highlighted,^[^ ^14^ ^]^ magnetic field orientation of nanofillers could be a versatile method for arbitrary orientation provided that a relatively high magnetic field could be generated in a compact orientation cell and if curvatures in magnetic field lines could be avoided, in order to reach a long‐range uniform orientation of nanofillers. Thus, drawing inspiration from advances in low‐field Nuclear Magnetic Resonance (NMR) technology^[^ ^15^ ^]^ and its potential for inline applications,^[^ ^16^, ^17^ ^]^ we have recently developed a novel orientation cell using a Halbach array.^[^ ^18^ ^]^ The Halbach array represents a unique arrangement of permanent magnets positioned to generate an extremely uniform and ultra‐high magnetic field within a specific area. The Halbach array designed for this purpose produces a magnetic field of 1 T with ultra‐high uniformity of magnetic field lines at the center core, which has a diameter of 30 mm and remarkably, very compact, with an overall diameter of 105 cm. We have demonstrated its aligning potential on graphene, carbon nanotubes, cellulose nanocrystals and wood fibers. The thermoelectrical properties had shown remarkable enhancements. In addition, a reduction of up to 50% of the filler content required to achieve antibacterial surfaces with efficacy on par with the benchmark in the field^[^ ^9^ ^]^ with the added benefit or arbitrary orientation.

In this study, we utilize the newly developed Halbach array orientation cell^[^ ^18^ ^]^ to generate a robust and uniformly distributed magnetic field, facilitating the alignment of hBN nanosheets within a PDMS matrix. We aim to explore the potential benefits of incorporating hBN, known for its exceptional thermal conductivity and electrical insulation properties. PDMS is selected as the matrix material due to its flexibility and ease of fabrication for coatings. Remarkably, the addition of Fe_3_O_4_ nanoparticles to hBN prevents agglomeration even within non‐polar polymers such as PDMS. Our hypothesis is that given the orientation conditions, superior thermal conductivities could be achieved in the nanocomposites. Thus, the primary objective of this study is to comprehensively analyze the influence of alignment achieved through static and rotating magnetic systems on hBN nanosheets' orientation within polymer matrices and to assess the resultant thermal properties. By elucidating these aspects, we seek to advance the understanding of hBN nanosheets‐polymer composite technology and its potential applications in various industries requiring enhanced thermal conductivity and other desirable properties. In summary, our research endeavors to address existing challenges in polymer thermal conductivity enhancement by introducing a novel approach that leverages magnetic field alignment with a Halbach array. Through systematic investigation and analysis, we aim to contribute to the advancement of polymer nanocomposite technology and its practical applications.

## 2 Experimental Section

### 2.1 Synthesis of Magnetic Boron Nitride

**Figure 1:**
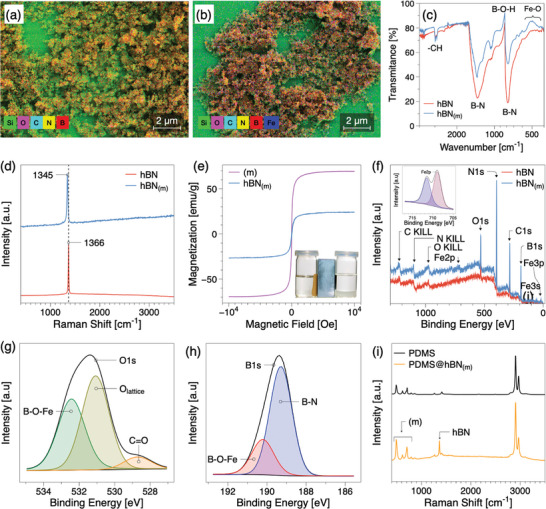
a) SEM and EDS image showing the morphology of the pure hBN sample revealing the chemical composition of a Si wafer. b) SEM and EDS results for hBN_(m)_ nanosheets, confirming the attachment of iron oxide. c) and d) FTIR and Raman spectra of hBN and hBN_(m)_. e) Magnetization hysteresis loop at room temperature, illustrating the magnetic characteristics of the investigated materials. The inset of (e) highlights the separation behavior of hBN_(m)_ in deionized (DI) water when exposed to an external magnetic field. f) show the XPS spectra of hBN and hBN_(m)_. The inset of (f) highlights the Fe 2p peak, confirming the successful doping of iron oxide on hBN to form hBN_(m)_ nanosheets. g) and h) show the XPS spectra of the O 1s and N 1s peaks of hBN_(m)_. i) Raman spectra of pure PDMS and PDMS@hBN_(m)_ composites, highlighting the structural differences.

To initiate the synthesis of magnetically responsive hBN nanosheets, Hexagonal boron nitride (hBN) nanosheets (density of 2.5 g cm^−3^) with a purity of 99.7% and an average size of 300 nm, procured from Nanografi Nano Technology in Turkey, was chosen as the primary material. The hBN nanosheets were first dispersed in deionized (DI) water at a concentration of 10 mg mL^−1^. Boron nitride (BN) nanosheets, being diamagnetic materials, typically require a magnetic field of at least 9 T to achieve alignment.^[^ ^14^ ^]^ However, generating and controlling such high magnetic fields was impractical for most industrial applications. To address this limitation, iron (Fe_3_O_4_) nanoparticles were utilized to reduce the magnetic field requirement to ≈1 T, a level that is more feasible and controllable.^[^ ^14^ ^]^ Hence, to impart magnetic properties to the 2D nanosheets, a procedure recently elaborated by Ghai et. al (2024) was used.^[^ ^18^ ^]^ Thereby, the dispersion underwent functionalization with a cationic‐type ferrofluid (EMG 605, Ferrotech, USA) through electrostatic adsorption due to the presence of negative charges, which attract the positively charged iron nanoparticles. hBN nanosheets doped with the magnetic nanoparticles were referred as _(m)_. The ferrofluid concentration for this study was kept constant at 0.003% for fabrication of Fe‐doped hBN nanosheets. The Zeta potential of cationic Fe_3_O_4_ and negatively charged hBN nanosheets at pH 7.0 had ≈+28 mV and ≈−36 mV, respectively. This large difference in charge (Δξ = 64 mV), only slightly lower than it was previously obtained using on graphene oxide.^[^ ^18^ ^]^ The high zeta potential of approximately ≈−39 mV for hBN_(m)_ at pH 7.0 results in exceptionally robust attachment of iron particles to boron nitride nanosheets, showing no discernible impact from sonication and high‐shear mixing. The visually distinctive light brown powder (hBN_(m)_) sediments were then subjected to an oven for a slow drying up at 40 °C for 24 h to ensure the elimination of water present to yield the final magnetic‐responsive hBN_(m)_ powder. The presence of iron particles with dimensions ranging from 10 to 15 nm on hBN is further confirmed by SEM and EDS, shown in **Figure** **1**a,b. It can be evidenced by TEM images in Figure S1 (Supporting Information) that the hBNs have an average diameter of 300 nm with an average thickness of 3 nm (aspect ratio 100). Upon doping the hBN nanosheets with Fe nanoparticles, they become attached, as depicted in Figure S1 (Supporting Information).

### 2.2 Composite Fabrication

For detailed studies, magnetically responsive boron nitride nanosheets hBN_(m)_ were prepared at a concentration of 10 wt.% (4.11 vol.%) and added them to a mixture of polydimethylsiloxane (PDMS) base and curing agent in a 10:1 ratio, using materials from Dow Corning Sylgard 184 Silicone Elastomer. The mixture was thoroughly stirred for 5 min on a magnetic stirrer to ensure uniform blending of the curing agent, resin, and nanoparticles. To eliminate any remaining agglomerates, the mixture was then sonicated for 15 min, resulting in a uniformly dispersed hBN_(m)_ in the polymer matrix. The resulting solution was carefully poured into a mold and then subjected to alignment within a Halbach array under either a static or rotating magnetic field for 10 min. Then, the composite was left to cure at room temperature for 24 h, after which it was removed and cured at 80 °C for an additional 5 h to ensure the complete curing of the fabricated composite.^[^ ^19^, ^20^ ^]^ All experiments in the current study were repeated three times to ensure the reliability and consistency of the results. Samples with a maximum length of 4 cm were fabricated using this process.^[^ ^18^ ^]^ The orientation within these samples was remarkably uniform throughout, attributed to the highly consistent magnetic field generated by the Halbach array.

### 2.3 Characterization

The surface morphology of prepared composites and the size of boron nitride nanosheets and Fe_3_O_4_ nanoparticles were analyzed in a secondary electron (SE) mode using LEO 1550 Gemini field emission gun SEM (FEG‐SEM).

Raman spectra were recorded on a WITec alpha300 R Raman spectrometer with a 532 nm laser line. For Raman analysis, powder samples were placed on the surface of the glass slide and firmly pressed for a homogeneous consistency.

Fourier‐transform infrared spectroscopy (FTIR) was performed using a PerkinElmer Spectrum One (Connecticut, USA) instrument equipped with an Attenuated Total Reflectance (ATR). A background of air was collected prior to the measurements, and the samples were placed on top of the ATR crystal and secured using a metal clamp to ensure consistent pressure. The device was set to collect the spectra in transmittance mode between 4000 and 400 cm^−1^, with 32 scans being collected with a resolution of 2 cm^−1^.

XPS analysis was performed using a PHI 5000 VersaProbe III Scanning XPS Microprobe (Physical Electronics, Feldkirchen, Germany) at an angle of 45°. The hBN nanosheets with and without Fe_3_O_4_ nanoparticles were drop casted on the silicon wafers and subjected to the XPS analysis.

Zeta potential measurements of hBN nanosheets and Fe_3_O_4_ magnetic nanoparticles (m_*p*_) suspensions were performed using a Zetasizer Nano ZS (Malvern Instruments, UK) with DTS1070 folded capillary cells. Both the suspensions were diluted to 0.01 wt%, and all the measurements were conducted at 23 °C, with a stabilization time of 120 s, repeated three times, and the average value was reported.

A Princeton Measurements Corp. MicroMagTM 2900 alternating gradient magnetometer (AGM) was used to characterize the magnetic properties of the sample. Prior to measurements, samples were prepared on the silicon (Si) substrate. The substrates were weighed both before and after the deposition of samples to determine the exact weight of the samples with a precision of up to four decimal places. The samples prepared on the Si substrates were attached to the in‐plane probe attachment using vacuum grease. Magnetization curves were obtained through in‐plane field sweep mode from 10^4^ to −10^4^ Oe at room temperature. The built‐in linear slope correction was performed after the measurement.

An Anton Paar MCR702e Space rheometer (Graz, Austria) was used in single motor transducer configuration to study the rheological behavior of composite and its curing kinetics. Rheological measurements were performed using a parallel plate measuring system with a diameter of 25 mm (PP25) and a P‐PTD 200 accessory was used to maintain the measuring temperature at 23 °C. The oscillatory strain sweep test was performed at a constant strain amplitude of 0.002% and angular frequency of 6.28 rad s^−1^, and the evolution of storage and loss moduli (*G*′ and *G*″ respectively) was measured with time. Based on the identified linear viscoelastic region, frequency sweep tests were performed at a constant strain amplitude of 10%.^[^ ^18^ ^]^

A Novocontrol Alpha (Montabaur, Germany) spectrometer was used to determine the dielectric spectra and subsequently the DC conductivity in the limit of low frequencies, σ_*DC*_ = lim_*f* → 0_σ′(*f*), where σ′ is the real part of the electrical conductivity, as determined from the imaginary part of the dielectric permittivity.

Bright‐field TEM (BF‐TEM) images were collected on an FEI Tecnai T20 microscope (FEI, USA), equipped with a LaB6 filament and operating at 200 keV acceleration voltage. Both hBN and hBN_(m)_ samples were deposited on commercial electron‐transparent substrates consisting of a holey‐carbon film on a copper grid (Ted Pella, Inc).

Thermal conductivity was measured using Hot Disk TPS 2500S thermal analyzer (Hot Disk AB, Göteborg, Sweden). The measurement proceeds by selecting the appropriate heating power, measurement time and sensor type. For the current work, a sensor with a radius of 0.526 was used for all the measurements. All the measurements were carried out at room temperature.

## 3 Results and Discussion

To probe into the chemical functionalization of prepared hBN_(m)_, Fourier Transform Infrared Spectroscopy (FTIR) analysis were conducted, and the spectra of hBN and hBN_(m)_ are compared in Figure 1c. Both hBN nanosheets and hBN_(m)_ exhibit several oxygen‐bearing functional groups. The characteristic peaks of hBN, namely the B─N bonds, are clearly observed at 783 and 1376 cm^−1^. The former corresponds to the out‐of‐plane vibration of the B─N─B bonds, while the latter represents the in‐plane stretching vibration of the B─N transverse optical mode of sp^2^ bonded hexagonal boron nitride (hBN).^[^ ^21^ ^]^ Additionally, a peak at 1100 cm^−1^ corresponding to B─O─H in‐plane bending is observed, along with an additional functional group peak at ≈2900 cm^−1^ corresponding to ─CH stretching.^[^ ^22^ ^]^ Notably, in the case of hBN_(m)_, an additional peak is observed at 578 and 440 cm^−1^, confirming again the successful attachment of Fe_3_O_4_ m_*p*_ onto hBN nanosheets. The Raman spectra of hBN in Figure 1d exhibit a sharp peak at 1366 cm^−1^ attributed to the E2g phonon mode, corresponding to the B─N vibration mode.^[^ ^23^, ^24^ ^]^ This peak bears similarity to the G‐band observed in graphene. Upon the attachment of m_*p*_ to hBN, a significant peak shift of 22 cm^−1^ is observed. This shift can be attributed to the introduction of the impurity m_*p*_ onto the pristine hBN nanosheets, confirming the successful attachment of m_*p*_ onto the hBN nanosheets and the formation of hBN_(m)_. The attachment of m_*p*_ onto hBN nanosheets is anticipated to introduce magnetism into the hBN nanosheets, allowing them to orient in a magnetic field of 1 T when dispersed within a polymer matrix. To confirm the presence of magnetic properties, saturation magnetization measurements were conducted for both m_*p*_ and hBN_(m)_. It can be seen that due to the size of the m_*p*_ nanoparticles, ≈10–15 nm, they exhibit superparamagnetic behavior with a saturation magnetization of 69 emu g^−1^, see Figure 1e. Furthermore, hBN_(m)_ exhibits a saturation magnetization of 24 emu g^−1^, ≈44% lower compared to the saturation magnetization obtained using reduced graphene oxide.^[^ ^18^ ^]^ While we have not fully elucidated the rheological parameter space for orientation efficiency in the highly uniform magnetic field conditions of the newly developed Halbach array orientation cell, this suggests that the optimal rheological conditions elaborated in our previous work would need to be re‐considered when using hBN Additionally, hysteresis loops indicate that both m_*p*_ and hBN_(m)_ are superparamagnetic, indicating that they would not agglomerate due to residual magnetism when the external magnetic field is removed. The inset image in Figure 1e demonstrates the magnetic properties of hBN_(m)_, exhibiting a magnetic response to an external magnetic field compared to the non‐magnetic hBN nanosheets. Moreover, we note that the high magnetic properties of the filler were achieved with an extremely low m_*p*_ wt.% (0.003). We carefully chose a low Fe concentration to maintain the integrity of hBN's intrinsic properties while still achieving alignment using a magnetic field. While this lower concentration necessitated a higher magnetic field (1 T) to accomplish alignment, it allowed us to preserve the thermal and structural characteristics of hBN more effectively than higher Fe‐doped systems reported in previous research. This approach ensures that the unique benefits of hBN, such as its high thermal conductivity and chemical stability, are largely retained in our composites. Notably, a defect concentration as low as 0.3% in hBN can reduce thermal conductivity by 40%, while a 0.9% concentration can lead to a 60% reduction. Thus, by minimizing Fe incorporation, we mitigate defect formation, optimizing thermal conductivity and preserving the superior performance characteristics of hBN.^[^ ^25^, ^26^ ^]^

XPS analysis in Figure 1f reveals the presence of boron, nitrogen, and oxygen, with characteristic peaks observed at 190 eV (B1s), 397 eV (N1s), and 532 eV (O1s), respectively.^[^ ^27^ ^]^ Notably, in the pristine hBN nanosheets, weak peaks of carbon and oxygen are also detected. The C1s peak is attributed to the binding energy of carbon contaminants, while the O1s peak is associated with oxygen atoms present at the edge plane of the pristine hBN nanosheets. Upon attachment of Fe_3_O_4_ onto the hBN nanosheets through electrostatic interactions, a new peak corresponding to Fe_3_O_4_ is observed in the scan. Further analysis via a narrow scan (see insert of Figure 1f) reveals the presence of Fe_3_O_4_ and confirms once again its successful attachment onto the hBN nanosheets, forming hBN_(m)_. This is based on the peaks observed in both the survey and narrow scan at 711.5, 94, and 56 eV that can be attributed to Fe2p3/2, Fe3s, and Fe3p, respectively.^[^ ^28^ ^]^ In a detailed scan of oxygen (O1s) in Figure 1g, it is observed that the binding energy value of 532.9 eV corresponds to lattice oxygen, 534.6 eV corresponds to the B─O─Fe chemical bond, and 528.7 eV corresponds to the C═O chemical bonding.^[^ ^29^ ^]^ Similarly, a detailed scan of boron (see, Figure 1h) also reveals the presence of B─N and B─O─Fe bonding at peak positions of 189.3 and 190.2 eV, respectively.^[^ ^30^ ^]^ Further, Raman spectroscopy (Figure 1i) validates the presence of hBN_(m)_ nanosheets in the PDMS composite, as evidenced by characteristic peaks corresponding to both hBN_(m)_ and PDMS.

To reach the full potential of hBN nanosheets as thermally enhancing additives in polymer composites, it is essential to orient hBN_(m)_.

**Figure 2:**
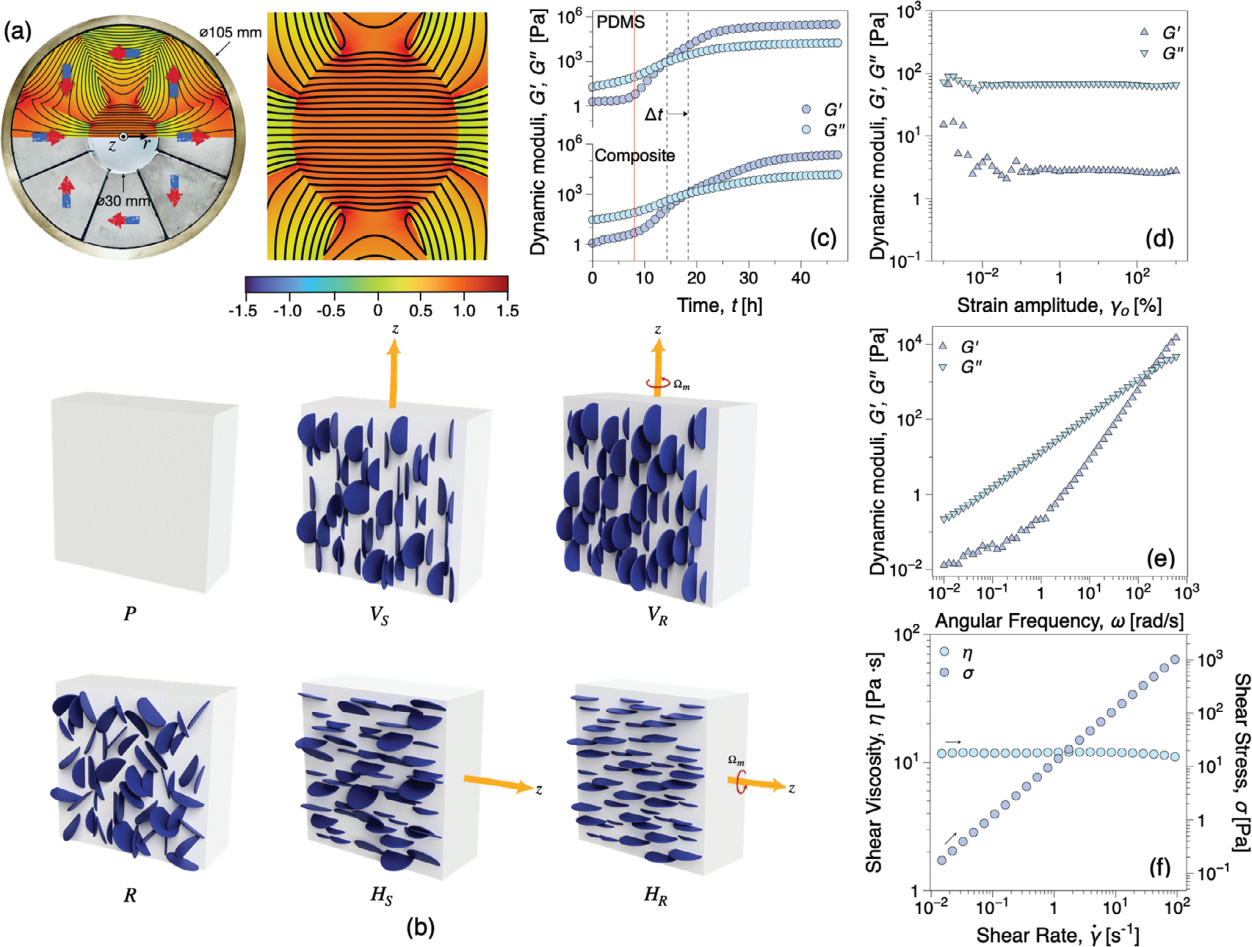
a) Halbach Array represented with the numerical model showing the magnetic field in one half of the axis of symmetry, alongside a zoomed‐in view of the entire Halbach magnet array core. b) Schematic representation of various orientations in the composite when subjected to static or rotating magnetic fields: P represents pure PDMS, R represents random orientation, and H and V represent horizontal and vertical orientations, with subscripts S and R indicating static and rotating magnetic fields applied from a Halbach array. c) Curing kinetics of PDMS and the composite with 10 wt.% hBN_(m)_. d–f) Amplitude sweep, frequency sweep, and viscosity measurements of PDMS and the composite containing hBN_(m)_, measured at t = 8 h, see solid red line in Figure 2c.

During experimentation, samples are positioned at the center of the core and exposed to the magnetic field (Halbach Array; Figure 1a for a duration of 10 min, leading to the alignment of hBN nanosheets in the direction of the applied magnetic field. The schematic in **Figure** **2**b shows the various nanocomposite morphologies investigated in this study: (P) ‐ PDMS (no fillers), (R) ‐ random orientation (no magnetic field), (H) ‐ “horizontal” orientation configuration with the magnetic field lines oriented in‐plane with the sample, (V) ‐ “vertical” orientation configuration with the orientation direction perpendicular with the sample plane. Indices _*S*_ and _*R*_ refer to static ($\Omega_{m}=0$) and rotating magnetic fields ($\Omega_{m}>600$ rpm; note: the sample is rotating while the magnet is stationary as shown in Figure S2).

The rheology of the surrounding matrix strongly influences the orientation dynamics of nanofillers, that is, the rate at which they align in a magnetic field.^[^ ^14^ ^]^ Therefore, understanding the rheological behavior is essential for orienting magnetic nanosheets (hBN_(m)_) in a matrix such as PDMS. To shed light on this, we studied the curing kinetics of both pure PDMS and the nanocomposites. Introducing PDMS@hBN_(m)_ into the magnetic field immediately after mixing results in rapid alignment. However, subsequent translation^[^ ^14^ ^]^ of the nanosheets toward the magnet occurs due to the composite's low viscosity, resulting in a highly inhomogeneous distribution of the nanofillers inside the matrix, see Figure S3 (Supporting Information). Thus hBN_(m)_ nanosheets must be introduced into the magnetic field at a partially cured state, which constitutes an optimal rheological window.^[^ ^18^ ^]^ For pure PDMS, *G*″ > *G*′ for the first 8 h of curing, after which a sharp increase of *G*′ is observed, marking the onset of gel formation, Figure 2c. Subsequently, at t = 14 h, *G*′ crosses over *G*″, indicating the formation of a gel‐like network in the PDMS matrix. After 14 h, *G*′ and *G*″ continue to increase and finally plateau at *t* = 26 h, marking the completion of the curing process. The gel‐like network of PDMS inhibits the alignment of hBN_(m)_ nanosheets. Therefore, PDMS@hBN_(m)_ was introduced in the magnetic field at t = 8 h, before the gel point. For comparison, the dynamic moduli evolution with time for the composite material is measured and it was observed that the *G*′, *G*″ crossover was delayed by ≈ 4 h upon the addition of hBN_(m)_ to the PDMS matrix, see Figure 2c. The strain sweep test in Figure 2d confirms that the composite material has not reached a gel‐like state (*G*″ > *G*′) for a large range of strain amplitudes. Figure 2e shows that a gel‐like behavior can be observed only at angular frequencies greater than 200 rad s^−1^. Furthermore, Figure 2f illustrates the shear viscosity and shear stress functions of the composite at t = 8 h.

**Figure 3:**
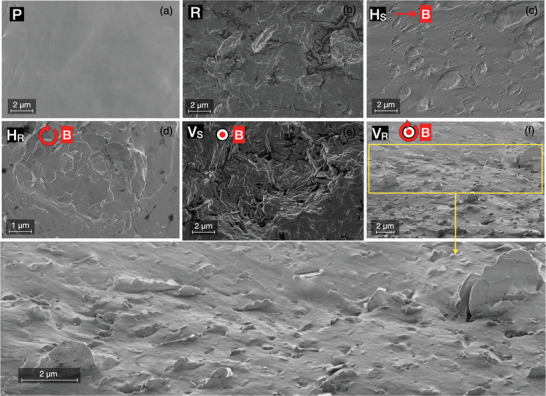
SEM images showing the surface morphology of the prepared samples: a) Pure PDMS, b) Random composite sample (without magnetic field), c) horizontally oriented composite sample in a static magnetic field, d) horizontally oriented composite sample in a rotating magnetic field, e) vertically oriented composite sample in a static magnetic field, and f) vertically oriented composite sample in a rotating magnetic field.

**Figure** **3** illustrates the surface morphology of various nanocomposites alongside pure PDMS prepared for this study. A smooth surface with fully crosslinked PDMS is observed in Figure 3a. For the composite, Boron Nitride nanosheets (hBN_(m)_) were incorporated into PDMS at a concentration of 10 wt.%. When hBN_(m)_ is added without any magnetic field, resulting in the Random (R) composite, nanosheets are observed to arrange randomly on the surface morphology, Figure 3b. However, when a magnetic field is applied parallel to the sample plane (Figure 3c,d), the hBN_(m)_ filler edges are entirely absent from the surface morphology of the nanocomposites, suggesting their preferential horizontal alignment. This is valid for both H_*S*_ and H_*R*_. A better distinction between the static and rotating magnetic cases can be made based on the vertical orientation cases, as their surface morphology is visualized perpendicular to the orientation direction. In Figure 3e, under the influence of V_*S*_ a non‐planar orientation with a clear network is evident. It is noteworthy that hBN_(m)_ nanosheets, possessing two degrees of freedom, orient themselves vertically under a magnetic field while retaining rotational freedom around their locked axis.^[^ ^14^ ^]^ To achieve planar orientation within PDMS, a rotating magnetic field ($\Omega_{m}>600$) was employed.^[^ ^18^ ^]^ After 30 min of rotation under a 1 T field, the hBN_(m)_ nanosheets achieved vertical alignment parallel to the rotating magnetic field, Figure 3f (V_*R*_). This arrangement is confirmed through a zoomed view showing the sequential alignment of the nanosheets along the magnetic field lines leading to a nearly perfect orientation of hBN_(m)_ nanosheets in the vertical direction (V_*R*_). The distinction between V_*S*_ and V_*R*_ can also be inferred from the H_*S*_ and H_*R*_, despite not being readily observable in the visualization plane.

### 3.1 Flexible and Actuation of Magnetic composite

**Figure 4:**
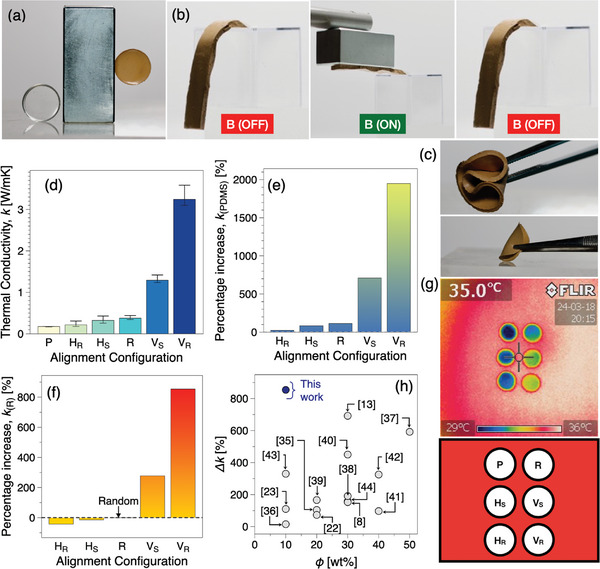
a–c) Demonstration of magnetic properties, a potential application in the field of magnetic actuation, and the inherent ultra‐flexibility of the PDMS@hBN_m_ nanocomposite d) Variation in thermal conductivity of PDMS and PDMS@hBN_(m)_ nanocomposites with different alignment configurations under a uniform magnetic field generated by a Halbach array. e,f) Percentage increase in thermal conductivity of PDMS@hBN_(m)_ composite compared to pure PDMS and randomly aligned samples. g) Infrared thermal images showcasing the heat transfer capabilities of aligned versus non‐aligned PDMS@hBN_(m)_ nanocomposites. h) Comparative percentage improvement in thermal conductivity when the aligned sample is compared to unaligned relative to similar studies.

Polydimethylsiloxane (PDMS) is a versatile elastomer known for its flexibility, transparency, and biocompatibility.^[^ ^19^, ^31^ ^]^ A defining characteristic of PDMS, like the vast majority of polymeric materials, is its inherent non‐magnetic nature, remaining unaffected by magnetic fields. However, when PDMS is combined with magnetically doped boron nitride nanosheets (hBN_(m)_) to form the nanocomposite PDMS@hBN_(m)_, a significant transformation occurs, endowing the resulting material with magnetic properties. This magnetism is showcased in **Figure** **4**a. The magnetic behavior of the PDMS@hBN_(m)_ nanocomposite arises from a combination of factors. First, the presence of hBN_(m)_ nanosheets within the PDMS matrix increases the material's susceptibility to external magnetic fields, leading to a noticeable attraction toward magnets. Second, the dispersion and alignment of hBN_(m)_ nanosheets within the PDMS matrix play a crucial role in influencing the composite's magnetic properties. Achieving a uniform distribution and alignment of hBN_(m)_ nanosheets enhances the magnetic response of the composite material. Further, to demonstrate the potential application of the fabricated magnetic PDMS@hBN_m_ nanocomposite, an experiment is conducted to observe its behavior under the influence of a magnetic field. As shown in Figure 4b, the magnetic field is applied to the nanocomposite, and its response is meticulously recorded. Initially, the nanocomposite exhibited no movement in the vertical direction when the magnetic field was absent. This lack of motion indicated the stability and passive nature of the material under normal conditions. However, upon the application of the magnetic field, a significant change in behavior is observed. This actuation occurs due to the embedded aligned magnetically responsive (hBN_(m)_) fillers in PDMS when responding to the magnetic field. The orientation of these nanofillers generates an anisotropic force within the PDMS matrix, effectively pulling the composite toward the magnetic source. The composite began to actuate in the vertical direction, moving upward toward the magnet positioned ≈15 mm above it. This movement clearly demonstrated the composite's responsiveness to the magnetic field. The actuation process continued until the composite reached the magnet, showing a strong attraction between the material and the magnetic field source. Once the magnetic field is removed, the composite promptly returns to its initial position. This reversible actuation highlights the material's potential for applications requiring precise and controlled movements, such as in soft robotics, actuators, and sensors. Additionally, the inherent ultra‐flexibility of the PDMS matrix combined with the magnetic properties of the hBN_m_ filler contributes to the overall performance of the composite. The flexibility allows the material to bend and conform to various shapes, enhancing its adaptability in different applications (Figure 4c). The magnetic actuation capability, on the other hand, provides a means for remote control and manipulation without direct physical contact. This experiment underscores the synergy between flexibility and magnetic responsiveness in the PDMS@hBN_m_ nanocomposite. Such materials hold promise for innovative applications where traditional rigid materials fall short. For instance, in biomedical devices, the ability to actuate and move in response to external magnetic fields can lead to less invasive procedures and more efficient drug delivery systems. In soft robotics, the combination of flexibility and remote actuation can result in more lifelike and adaptable robots capable of performing delicate tasks. However, the fabricated composite deviates in transparency, exhibiting a light brown hue attributed to the incorporation of 10 wt.% hBN_(m)_ nanosheets. This comprehensive understanding of the PDMS@hBN_(m)_ nanocomposite's magnetic, flexibility, and transparency properties provides valuable insights for its potential applications in various fields.

### 3.2 Thermal Properties

An enhanced thermal conductivity observed in the aligned PDMS@hBN_(m)_ composite could result from several factors, including i) directional control over phonon transport, ii) the establishment of a percolated network structure and iii) reduced phonon scattering.^[^ ^26^, ^32^, ^33^ ^]^

The thermal properties of both pure PDMS (P) and the prepared PDMS@hBN_(m)_ composite were analyzed under ambient conditions. Pure PDMS demonstrates a thermal conductivity of 0.175 W mK^−1^, consistent with values reported in the literature.^[^ ^34^ ^]^ Notably, the random sample (R), lacking magnetic field orientation, displays a significantly higher thermal conductivity of 0.375 W/mK, ≈114% greater than the pure PDMS sample (P), as shown in Figure 4d,e. PDMS@hBN_(m)_ composite when subjected to a static magnetic field, anisotropy emerges. Vertically aligned (V_*S*_) nanocomposites, where the magnetic field aligns with the heat flow direction, achieve a thermal conductivity of 1.42 W mK^−1^ an ≈710% improvement over pure PDMS (P), as shown in Figure 4e. Conversely, horizontally aligned (H_*S*_) nanocomposites demonstrate a more modest enhancement in thermal conductivity, with an increase to only 0.321 W mK^−1^, or ≈83% higher than pure PDMS (P). This comparative analysis of the random (R), vertical (V_*S*_), and horizontal (H_*S*_) alignments provides clear evidence that the alignment mechanism plays a pivotal role in determining the extent of thermal conductivity enhancement.

Samples produced by rotating magnetic field, with vertical (V_*R*_) and horizontal (H_*R*_) alignment exhibit thermal conductivities of 3.584 and 0.216 W mK^−1^, respectively, representing enhancements of ≈ 1950% and 23%. The exceptional improvement in the V_*R*_ sample highlights the dominance of directional control and the formation of a highly percolated network under the influence of the uniform rotating magnetic field generated by the Halbach array. Achieving higher levels of anisotropy, as can be achieved using a Halbach array, thus appears to be essential to further enhance the thermal conductivity of the nanocomposite. This innovative approach stands out prominently among similar studies,^[^ ^8^, ^13^, ^27^, ^28^, ^35^, ^36^, ^37^, ^38^, ^39^, ^40^, ^41^, ^42^, ^43^, ^44^ ^]^ achieving the highest improvement in thermal conductivity, as illustrated in Figure 4h. The comparison is conducted by analyzing the hBN weight percentage (wt.%) and its corresponding percentage increase in thermal conductivity for magnetically aligned hBN composites relative to random composites. The mechanisms contributing to the substantial enhancement in thermal conductivity observed in the aligned composite could thus be attributed to a combination of factors i–iii) already mentioned. First, the remarkable morphological anisotropy shown, directional control can be perhaps considered as a dominant factor. This directional control is particularly evident in vertically aligned (V_*R*_) nanocomposites, where the magnetic field and heat flow direction coincide, leading to a significant enhancement in thermal conductivity. When the hBN_(m)_ nanosheets are precisely oriented within the PDMS matrix under the influence of a magnetic field, they form well‐defined, ordered pathways for efficient phonon transfers. Phonons–quantized lattice vibrations responsible for thermal energy transport–propagate through materials by interacting with neighboring atoms or molecules, and the alignment facilitates this movement in a targeted direction. Second, the establishment of a percolated network of hBN_(m)_ nanosheets within the (V_*R*_) composite creates a continuous pathway for phonon propagation, minimizing thermal resistance at the interfaces between the nanosheets and the polymer matrix. This coherent network effectively bridges gaps between individual hBN_(m)_ nanosheets, promoting phonon transport along the aligned direction. The scanning electron microscopy (SEM) analysis also seems to suggest that V_*S*_, at least, consisted of a percolated network. This experimental observation aligns with the proposed mechanism of reduced interfacial thermal resistance due to the continuous network formed by the aligned nanosheets. In our previous investigations using an electrically conductive filler, electrical conductivity data suggested that also V_*R*_ samples may reach percolation at very low concentrations due to the aggregation of fillers into conductive pathways.^[^ ^18^ ^]^ Finally, the alignment reduces phonon scattering by increasing the mean free path of phonons along the aligned axis. The directional orientation provided by the magnetic field minimizes scattering in other directions, further boosting the thermal conductivity of aligned hBN_(m)_ nanosheets by channeling energy flow through these conductive pathways. Together, these mechanisms–directional control, network percolation, and reduced phonon scattering–synergistically enhance the thermal conductivity of the nanocomposite. Figure 4f supports this conclusion by showing that vertical alignment significantly outperforms horizontal alignment and random orientation, underscoring the anisotropic advantages introduced by magnetic field alignment.

To comprehensively assess the impact of alignment on heat flow, the prepared aligned samples were compared with the (R) sample. Figure 4f provides compelling evidence of the profound effects of alignment on thermal conductivity. Intriguingly, both static and rotating vertical alignment configurations exhibit remarkable enhancements in thermal conductivity compared to the random sample, with increases of ≈ 854% and 277%, respectively. This substantial improvement underscores the pivotal role of alignment in facilitating efficient heat transfer within the composite. Conversely, in the case of both static and rotating horizontally aligned samples, a contrasting trend emerges, with thermal conductivity experiencing reductions of ≈14% and 42%, respectively, compared to the (R) sample. This decline suggests that horizontal alignment impedes heat flow within the composite, indicative of the anisotropic behavior induced by the magnetic field. Furthermore, the preparation of a vertically oriented sample (V_*R*_) under a uniform rotating magnetic field, generated by a Halbach array, represents a significant advancement in the field of thermal conductivity enhancement. This explicit correlation between mechanisms and experimental results further highlights the potential for magnetic alignment to revolutionize thermal management technologies.

To additionally validate the thermal conductivity results of the nanocomposite with different orientations, a thermographic analysis was conducted using a handheld infrared camera. The study encompassed the examination of prepared (P), randomly oriented (R), and aligned samples. Each sample was positioned on a preheated hot plate set at 35 °C, and temperature changes are monitored over 120 s. The thermographic results mirrored the trends observed in the thermal conductivity data. Notably, the (V_*R*_) sample exhibited the highest temperature increase, reaching 34.3 °C within the 120 s timeframe. Comparatively, the (V_*S*_), (R), (H_*S*_), (H_*R*_), and (P) samples attained temperatures of 33.8, 33.5, 32.5, 31.9, and 29.9 °C, respectively, see, Figure 4g. These findings corroborate the thermal conductivity data, further affirming the effectiveness of magnetic field‐induced alignment as a dominant factor in enhancing heat transfer within the nanocomposite. The thermographic analysis provides valuable complementary evidence, supporting the reliability and validity of the observed thermal conductivity improvements in the aligned samples. Overall, the results show that the utilization of a uniform rotating magnetic field offers precise control over the orientation of hBN_(m)_ nanosheets within the composite, leading to unparalleled enhancements in thermal conductivity. This achievement not only demonstrates the effectiveness of magnetic field‐induced alignment in optimizing thermal properties but also underscores the potential for further advancements in thermal management technologies.

### 3.3 Dielectric Properties

BN is a unique material that combines excellent thermal conductivity with outstanding electrical insulation properties, making it highly valuable in various advanced applications. In particular, boron nitride is frequently used as an insulating material in electronics where efficient heat dissipation is crucial.^[^ ^45^, ^46^, ^47^ ^]^ It is useful in applications like electronic packaging, heat sinks, and thermal interface materials, where maintaining electrical insulation while efficiently managing heat is essential. Its high thermal conductivity allows it to effectively transfer heat away from sensitive electronic components, preventing overheating and maintaining optimal performance. Moreover, hBN nanosheets, having excellent properties such as high dielectric constant, low dielectric losses, stability at high temperatures, insulation, thermal conductivity, and chemical inertness, open up significant potential in the field of energy storage capacitors compared to other carbon‐based nanomaterials.^[^ ^48^, ^49^, ^50^, ^51^ ^]^

**Figure 5:**
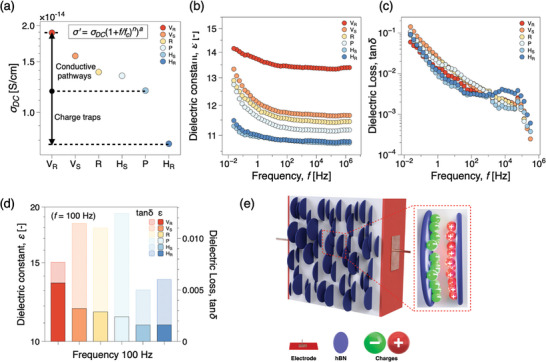
Comparative analysis of dielectric properties and DC conductivity in PDMS@hBN_(m)_ composites: a) DC conductivity σ_*DC*_, highlighting the formation of conductive pathways and charge traps, influenced by orientation configurations as determined from the dielectric spectra in Figure S4 (Supporting Information). b) Dielectric constant variation across samples shows orientation‐dependent capacitive behavior. c) Dielectric loss variation among samples at different frequencies, indicating the dissipation of electrical energy and highlighting differences in orientation configurations. d) Variation of dielectric constant and dielectric loss at a constant 100 Hz frequency showcasing the impact of orientation configurations on the composite's electrical properties under specific frequency conditions. e) Schematic showing the potential of aligned PDMS@hBN_(m)_ nanocomposite for energy storage capacitors due to its high dielectric constant, thermal conductivity, and insulating properties, along with low dielectric losses.

As observed in **Figure** **5**, when hBN_(m)_ nanosheets in PDMS are oriented in a magnetic field, they further enhance the dielectric constant and lower the dielectric losses. The dielectric properties of both pure PDMS and PDMS@hBN_(m)_ composites, both aligned and non‐aligned, are thoroughly examined across a frequency range from 0.0236 Hz to 2.73 MHz, as depicted in Figure 5. Analysis of the DC electrical conductivity data, showcased in Figure 5a, reveals the profound influence of alignment. Notably, while PDMS (P) exhibits an electrical conductivity of 1.2 × 10^−14^ S cm^−1^, this value increases slightly to 1.4 × 10^−14^ S cm^−1^ upon the incorporation of 10 wt.% randomly aligned hBN_(m)_ nanosheets. However, the introduction of alignment under a uniform magnetic field, facilitated by a Halbach array, results in no significant variation in electrical conductivity values among the aligned composites. Notably, the planar alignment of hBN_(m)_ nanosheets in a horizontal direction leads to a charge‐trapping effect within the composite, resulting in an insulating behavior. Consequently, (H_*R*_) exhibits an electrical conductivity of 0.77 × 10^−14^ S cm^−1^, representing approximately a 60%, 45%, and 35% decrease compared to (V_*R*_), (R), and (P), respectively. The enhanced insulating capability of aligned hBN_(m)_ nanosheets may be due to the few charge carriers available in the direction of electron flow. Thus, they act as a barrier to electron flow, increasing the overall resistance and reducing the conductivity of the composite. Additionally, this alignment forces the current to take a more convoluted path, further impeding electron movement. This characteristic makes (H_*R*_) ideal for applications requiring high thermal conductivity and effective electrical insulation. Moreover, (V_*R*_) demonstrates that the composite is still electrically insulating, reaching 1.9 × 10^−14^ S cm^−1^. The observed slight increase in the electrical conductivity within (V_*R*_) can be primarily attributed to several factors. These include the establishment of conductive pathways facilitated by enhanced interfacial interactions between hBN_(m)_ and PDMS. This phenomenon leads to reduced polarization relaxation and a high dielectric constant compared to other orientation configurations. Moreover, a similar trend is observed in the DC electrical conductivity data as observed in the thermal conductivity results. Specifically, the electrical insulation of aligned hBN_(m)_ nanosheets follows a distinct order: (H_*R*_) >(H_*S*_) >(R)>(V_*S*_) >(V_*R*_). Further analysis of the permittivity of PDMS and PDMS@hBN_(m)_ nanocomposites shows a significant dependence of the dielectric constant on the frequency from 0.0236 Hz up to 100 Hz, whereas there is no dependence on the frequency thereafter. The maximum value of the dielectric constant achieved with a planar orientation of hBN_(m)_ in a uniform rotating magnetic field (V_*R*_) is ≈14, which decreases to a value of 13.6 up to 100 Hz, after which it remains constant, as shown in Figure 5b.

To gain deeper insights into the impact of orientation on the dielectric properties, we compare the ε and dielectric loss of the PDMS and composite at 100 Hz. For a (V_*S*_) sample, the dielectric constant saturates at 11.8, which is 18.6% lower than (V_*R*_), as observed in Figure 5d. Moreover, the dielectric constant of the random sample increased very marginally by ≈2% compared to pure PDMS. Similar to electrical conductivity data, the horizontally aligned samples, both (H_*R*_) and (H_*S*_), have the lowest dielectric constant compared to all other samples; this might be due to the charge traps in these samples, with (H_*R*_) having the planar horizontal orientation exhibiting 24.12% and 6% lower dielectric constant values than (V_*R*_) and (R) at 100 Hz, as shown in Figure 5b,d. The dependence of dielectric loss on frequency is prominently illustrated in Figure 5c, wherein all samples exhibit a notable frequency dependency, with maximum dielectric losses observed at low frequencies. However, as frequency increases, dielectric losses decrease gradually. Remarkably, even with the addition of hBN_(m)_ to PDMS, dielectric losses remain unchanged, underscoring the material's potential across various electronic, electrical, and energy storage capacitor applications. Furthermore, Figure 5d provides insights into the dielectric losses of PDMS and PDMS@hBN_(m)_ composites with different orientations at 100 Hz. PDMS exhibits the highest dielectric losses at 0.012, followed by (V_*S*_) > (R) > (V_*R*_) > (H_*R*_) > (H_*S*_) with values of 0.0114, 0.0110, 0.0076, 0.0060, and 0.0049, respectively. Notably, the variation in dielectric losses across all samples is minimal, indicating a high level of consistency. These findings underscore the significant impact of alignment on the dielectric properties of the composite, highlighting the potential for tailored dielectric behavior through precise control of alignment configurations. The data show that vertically aligned samples, particularly (V_*R*_), still possess very high insulating properties with extremely high dielectric constants and low dielectric losses, making them potential candidates for use in applications such as energy storage capacitors as shown in Figure 5e. Moreover, the observed trends provide valuable insights for the design and optimization of polymer composites for advanced electronic applications, particularly in electronic packaging, heat sinks, and thermal interface materials

## 4 Perspectives on Highly Filled hBN Composites

Highly filled composites incorporating hexagonal boron nitride (hBN) nanosheets within polymer represent a significant leap in material science due to enhanced thermal conductivity and controlled electrical properties. The incorporation of hBN nanosheets into PDMS has led to a substantial improvement in thermal conductivity, with composites exhibiting up to 3.58 W mK^−1^, a 1950% and 854% increase compared to pure PDMS and unaligned composite, respectively. This enhancement is attributed to the alignment of hBN nanosheets under a uniform magnetic field, optimizing thermal pathways. Simultaneously, these composites maintain excellent electrical insulation, which is essential for electronic applications. Moreover, these BN‐based composites show significant promise in energy storage applications, particularly in capacitors. Vertically aligned hBN_(m)_ nanosheets in a rotating magnetic field achieve a dielectric constant of ≈14 and minimal dielectric losses of 0.0049 at 100 Hz, indicating their suitability for energy storage. Despite advancements, challenges remain in optimizing mechanical strength and scalability. Efficient processing techniques and improved curing methods are crucial for uniform material properties and scaling production. Future research should focus on balancing thermal, electrical, and mechanical properties, addressing long‐term stability, and exploring commercial applications in electronics, aerospace, automotive, biomedical fields, and energy storage. Ongoing innovation is essential for realizing the full potential of highly filled composites in high‐performance environments.

## Conflict of Interest

The authors declare no conflict of interest.

## Supporting information

Supporting Information

## Data Availability

The data that support the findings of this study are available from the corresponding author upon reasonable request.
