# Supplementary material for: Halbach Array Induced Magnetic Field Alignment in Boron Nitride Nanocomposites
Source: Adv Sci (Weinh). 2024 Dec 23;12(6):2408532. doi: 10.1002/advs.202408532 (PMC11809381; doi:10.1002/advs.202408532)
Supplement: Supplementary file 1 — Supporting Information [file ADVS-12-2408532-s001.pdf]

ADVANCED SCIENCE
Open Access

Supporting Information

for *Adv. Sci.*, DOI 10.1002/advs.202408532

Halbach Array Induced Magnetic Field Alignment in Boron Nitride Nanocomposites

Viney Ghai, Ases Akas Mishra, Enling Huang, Roselle Ngaloy, Saroj P. Dash, Aleksandar Matic and Roland Kádár**

Supporting Information

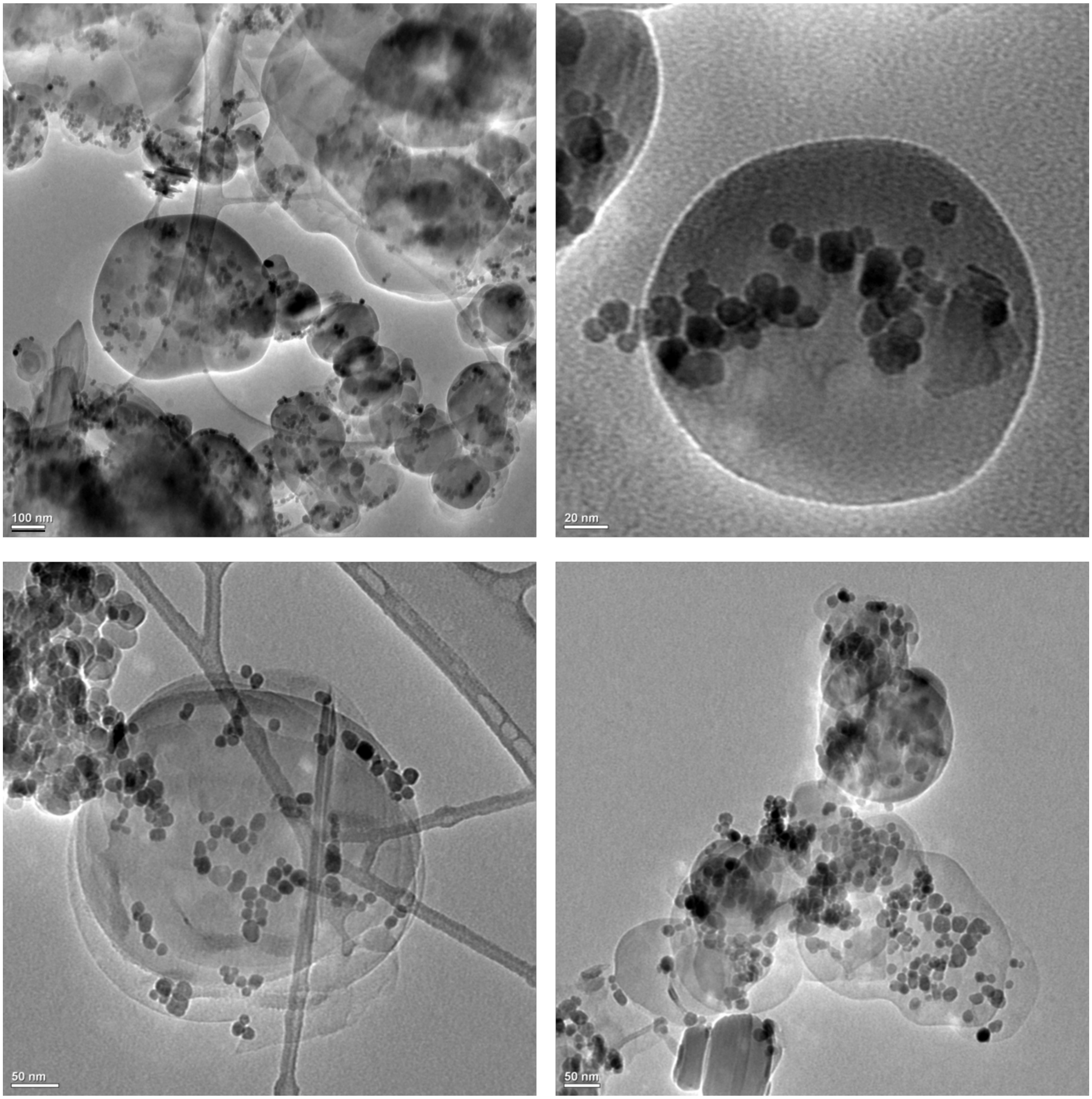

Figure S1. TEM image showing the adsorption of positively charged Fe_3O_4 nanoparticles onto negatively charged hBN nanosheets

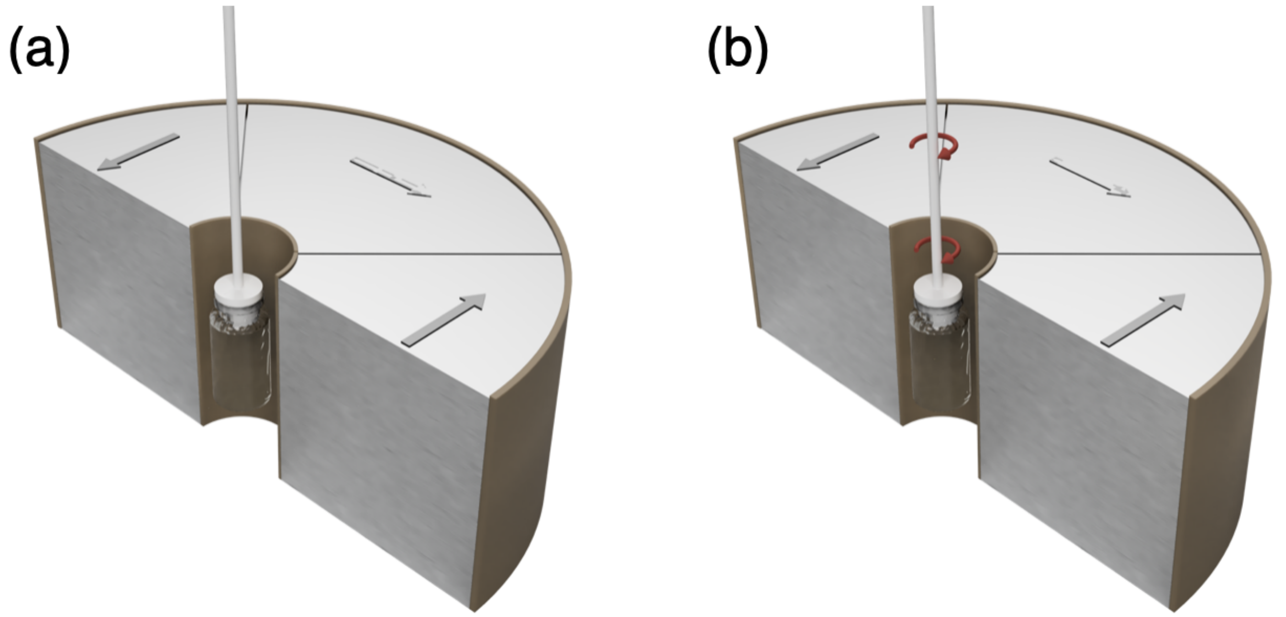

Figure S2. Schematic of hBN-polymer composite alignment using a Halbach array. (a) In a static magnetic field, hBN nanosheets align along the field direction with the sample held stationary within the array. (b) Under a rotating magnetic field, the sample rotates while the array remains static, resulting in planar alignment of hBN nanosheets. This configuration promotes structural ordering and percolation pathways, enhancing thermal conductivity.

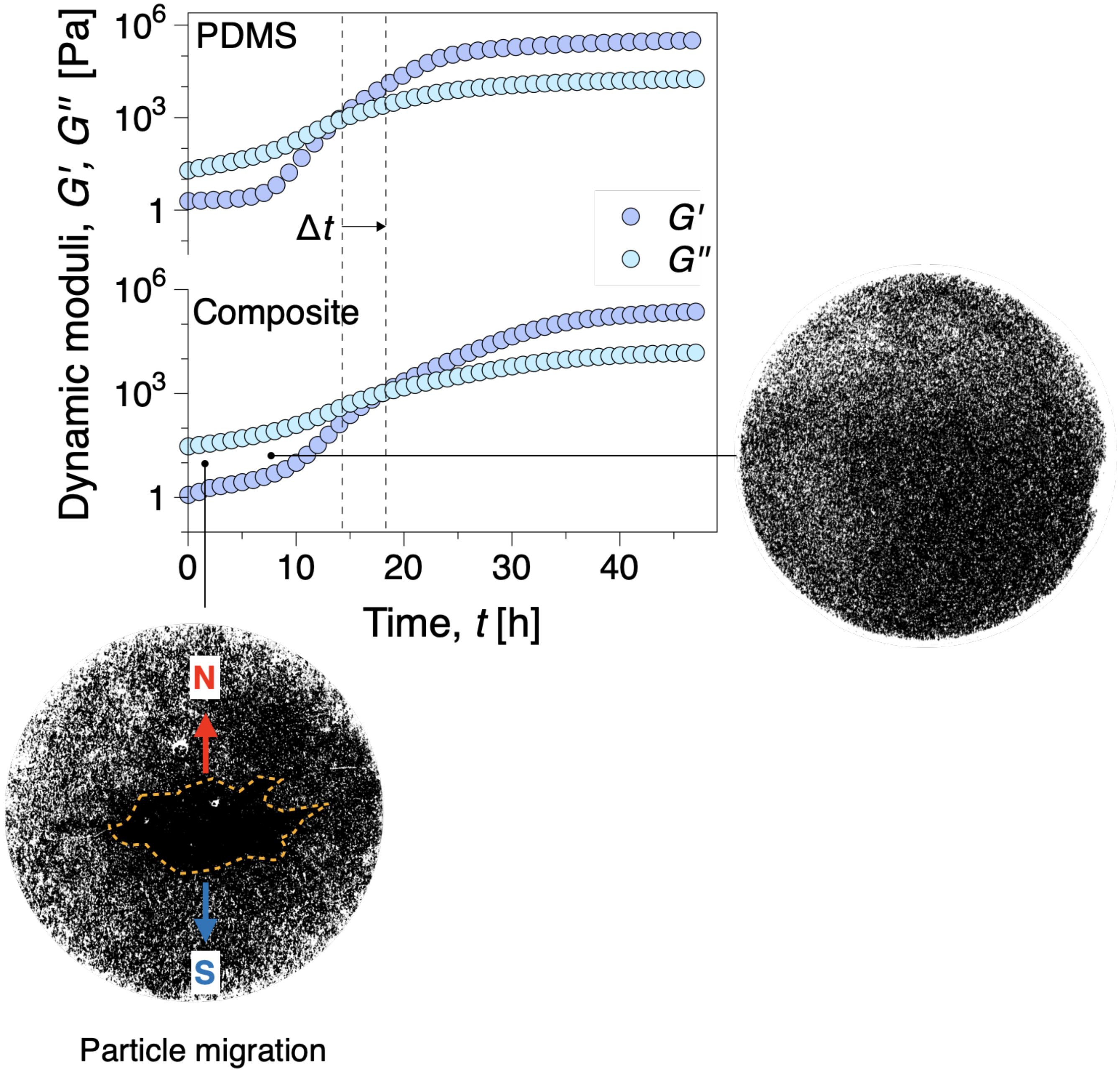

Figure S3. Figure showing the critical rheological window for aligning $hBN_{(m)}$ nanosheets in PDMS under a constant 1 Tesla uniform magnetic field.

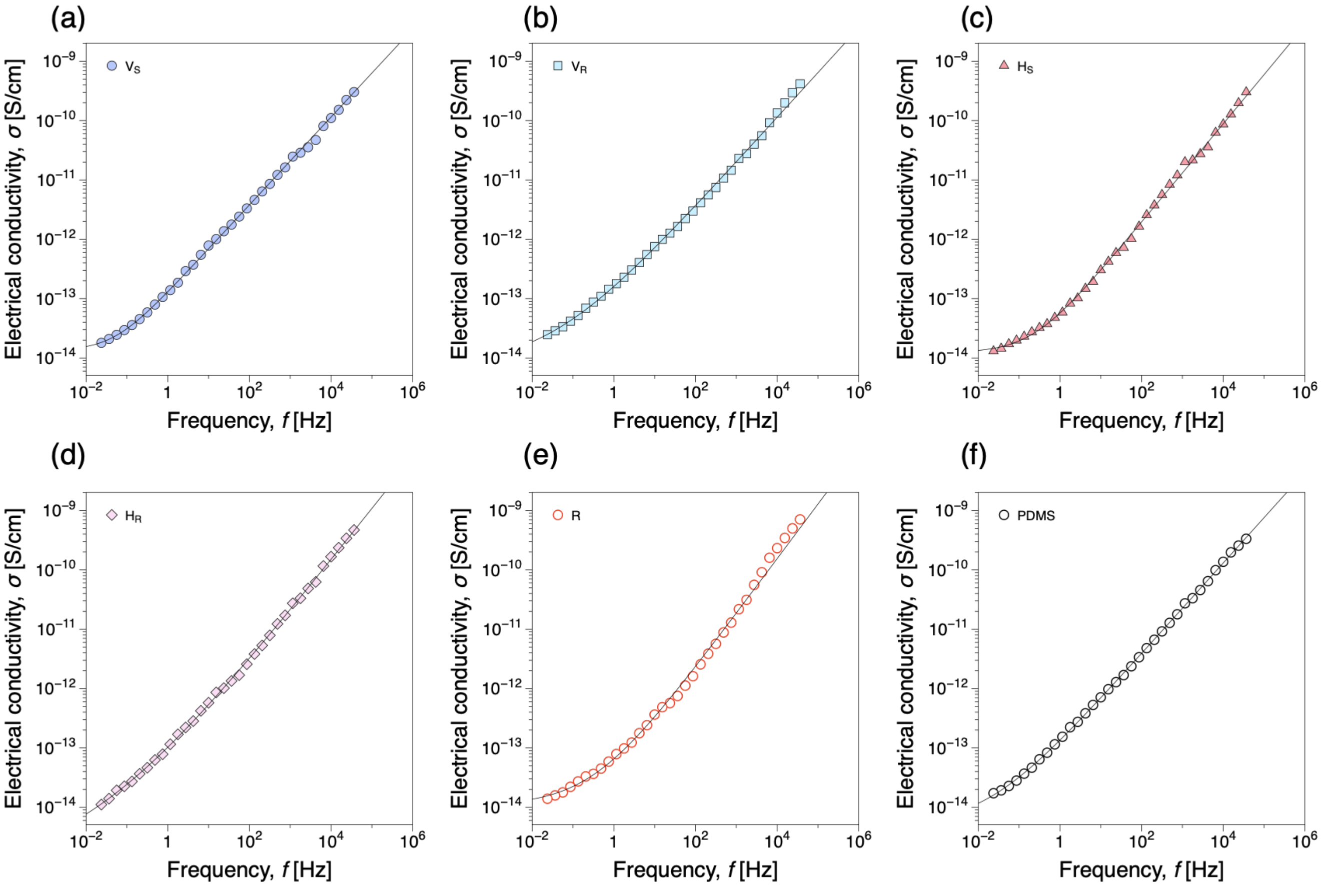

Figure S4. The figure depicts the variation in electrical conductivity among samples at different alignment configurations (a) (V_S) (b) (V_R) (c) (H_S) (d) (H_R) (e) R (f) Pure PDMS
